# Oral antiviral treatments for COVID-19: opportunities and challenges

**DOI:** 10.1007/s43440-022-00388-7

**Published:** 2022-07-25

**Authors:** Laila Rahmah, Sunny O. Abarikwu, Amanuel Godana Arero, Mickael Essouma, Aliyu Tijani Jibril, Andrzej Fal, Robert Flisiak, Rangarirai Makuku, Leander Marquez, Kawthar Mohamed, Lamin Ndow, Dorota Zarębska-Michaluk, Nima Rezaei, Piotr Rzymski

**Affiliations:** 1 School of Medicine, Tehran University of Medical Sciences, Tehran, Iran; 2 Universal Scientific Education and Research Network (USERN), Jakarta, Indonesia; 3 Department of Biochemistry, University of Port Harcourt, Choba, Nigeria; 4 Universal Scientific Education and Research Network (USERN), Choba, Nigeria; 5 Cardiac Primary Prevention Research Center, Cardiovascular Diseases Research Institute, Tehran University of Medical Sciences, Tehran, Iran; 6 Universal Scientific Education and Research Network (USERN), Addis Ababa, Ethiopia; 7 Department of Internal Medicine and Specialties, Faculty of Medicine and Biomedical Sciences, University of Yaoundé I, Yaoundé, Cameroon; 8 Universal Scientific Education and Research Network, Yaoundé, Cameroon; 9 Department of Community Nutrition, School of Nutritional Sciences and Dietetics, Tehran University of Medical Sciences, Tehran, Iran; 10 Nutritional and Health Team (NHT), Universal Scientific Education and Research Network (USERN), Tehran, Iran; 11 Universal Scientific Education and Research Network (USERN), Accra, Ghana; 12 Department of Population Health, Division of Public Health, Wroclaw Medical University, Wroclaw, Poland; 13 Collegium Medicum, Warsaw Faculty of Medicine, Cardinal Stefan Wyszyński University, Warsaw, Poland; 14 Integrated Science Association (ISA), Universal Scientific Education and Research Network (USERN), Poznań, Poland; 15 Department of Infectious Diseases and Hepatology, Medical University of Białystok, Białystok, Poland; 16 Universal Scientific Education and Research Network (USERN), Harare, Zimbabwe; 17 College of Social Sciences and Philosophy, University of the Philippines Diliman, Quezon City, Philippines; 18 Education and Research Network (USERN), Universal Scientific, Quezon City, Philippines; 19 Universal Scientific Education and Research Network (USERN), Manama, Bahrain; 20 National Health Laboratory Service, Kotu, Gambia; 21 Universal Scientific Education and Research Network (USERN), Banjul, Gambia; 22 Department of Infectious Diseases, Jan Kochanowski University, Kielce, Poland; 23 Research Center for Immunodeficiencies, Children’s Medical Center, Tehran University of Medical Sciences, Tehran, Iran; 24 Department of Immunology, School of Medicine, Tehran University of Medical Sciences, Tehran, Iran; 25 Network of Immunity in Infection, Malignancy and Autoimmunity (NIIMA), Universal Scientific Education and Research Network (USERN), Tehran, Iran; 26 Department of Environmental Medicine, Poznan University of Medical Sciences, Poznań, Poland

**Keywords:** SARS-CoV-2, Pandemic, Pharmaceutical treatment, Antivirals, Clinical trials

## Abstract

The use of antiviral COVID-19 medications can successfully inhibit SARS-CoV-2 replication and prevent disease progression to a more severe form. However, the timing of antiviral treatment plays a crucial role in this regard. Oral antiviral drugs provide an opportunity to manage SARS-CoV-2 infection without a need for hospital admission, easing the general burden that COVID-19 can have on the healthcare system. This review paper (i) presents the potential pharmaceutical antiviral targets, including various host-based targets and viral-based targets, (ii) characterizes the first-generation anti-SARS-CoV-2 oral drugs (nirmatrelvir/ritonavir and molnupiravir), (iii) summarizes the clinical progress of other oral antivirals for use in COVID-19, (iv) discusses ethical issues in such clinical trials and (v) presents challenges associated with the use of oral antivirals in clinical practice. Oral COVID-19 antivirals represent a part of the strategy to adapt to long-term co-existence with SARS-CoV-2 in a manner that prevents healthcare from being overwhelmed. It is pivotal to ensure equal and fair global access to the currently available oral antivirals and those authorized in the future.

## Introduction

The coronavirus disease 2019 (COVID-19), caused by severe acute respiratory system coronavirus 2 (SARS-CoV-2), was reported for the first time at the end of December 2019 and declared a pandemic by the World Health Organization by March 2020 [1]. The global viral spread has forced authorities to impose lockdowns, recommend various sanitary measures, and pursue diagnostic testing on a daily basis. The COVID-19 pandemic has rapidly overwhelmed the healthcare system, led to an economic crisis, and numerous changes in different strata of life [2–5]. During the first year, nearly 1.9 million deaths were attributed to COVID-19, followed by additional 3.55 million deaths reported in 2021 [6]. However, it is suggested that these figures may be largely underestimated, with global excess deaths two-fold or even four-fold higher during 2020–2021 [7].

The unprecedented response of the scientific community to the pandemic has led to rapid discoveries on SARS-CoV-2 viral entry and pathogenicity [8, 9]. A number of pharmaceuticals, such as chloroquine, hydroxychloroquine, darunavir, arbidol, favipiravir, lopinavir, remdesivir, ribavirin, ritonavir, interferons, dexamethasone, and tocilizumab, has been repurposed for treatment of COVID-19 with mixed results in clinical settings [10–14]. High hopes related to the use of convalescent plasma have been abandoned due to the lack of clinical benefits found in severely ill patients [15–17]. The clinical benefits of monoclonal antibodies targeting the SARS-CoV-2 spike protein have been proven in COVID-19 treatment [18], although their efficacy can be dramatically impacted by the currently circulating viral variant [19]. Nevertheless, the most significant disadvantage of various COVID-19 therapies was an inability to implement them outside the clinical setting. Depending on circumstances, the time between symptoms onset and hospitalization can exceed 10 days [20]. At that time, an immunocompetent patient with COVID-19 will no longer harbor the contagious virus, and instead of antiviral therapy, the management of respiratory distress, hyperinflammatory state, thrombosis, and other COVID-19 complications will be required [21, 22]. The main challenge of antiviral therapies is that they require implementation as soon as possible after acquiring the infection to act directly on viral replication—delayed administration of antivirals may result in a lack of effectiveness of such therapy [23, 24].

The authorization of various COVID-19 vaccines has brought hopes for better control of the spread of SARS-CoV-2 [25, 26]. However, clinical and post-authorization, real-world observations indicate that evidence that protection against infection gradually declines as serum antibody level decreases a few months after the last dose, whereas the circulation of highly transmissible variants such as Delta (B.1.617.2) and Omicron (B.1.1.529) may further increase the risk of contracting SARS-CoV-2 by vaccinated individuals [27–31]. In addition, Omicron lineage reveals a substantial immune escape from vaccine-acquired humoral immunity, further increasing the risk of breakthrough infections [32–34]. Therefore, the primary goal of COVID-19 vaccination is to decrease the rates of hospitalizations, admission to intensive care units, and deaths [35, 36]. Although this goal is often achievable due to the extended duration of vaccine-induced cellular immunity and can be further enhanced by the booster doses [37–40], certain vaccinated individuals may still experience severe COVID-19 due to a worse response to immunization. This can be due to elderly and age-related immunosenescence, primary or secondary immune deficiencies, and various lifestyle factors [27, 41–43]. All in all, maintaining high levels of protection from future waves of SARS-CoV-2 infections may require additional doses of first-generation or variant-adapted (preferentially multivariant) vaccines, or a novel approaches to vaccination. However, one should bear in mind that various populations may present a significant level of vaccine hesitancy or may be unable to continue vaccination due to side effects experienced following the previous dose [44–49].

**Fig. 1:**
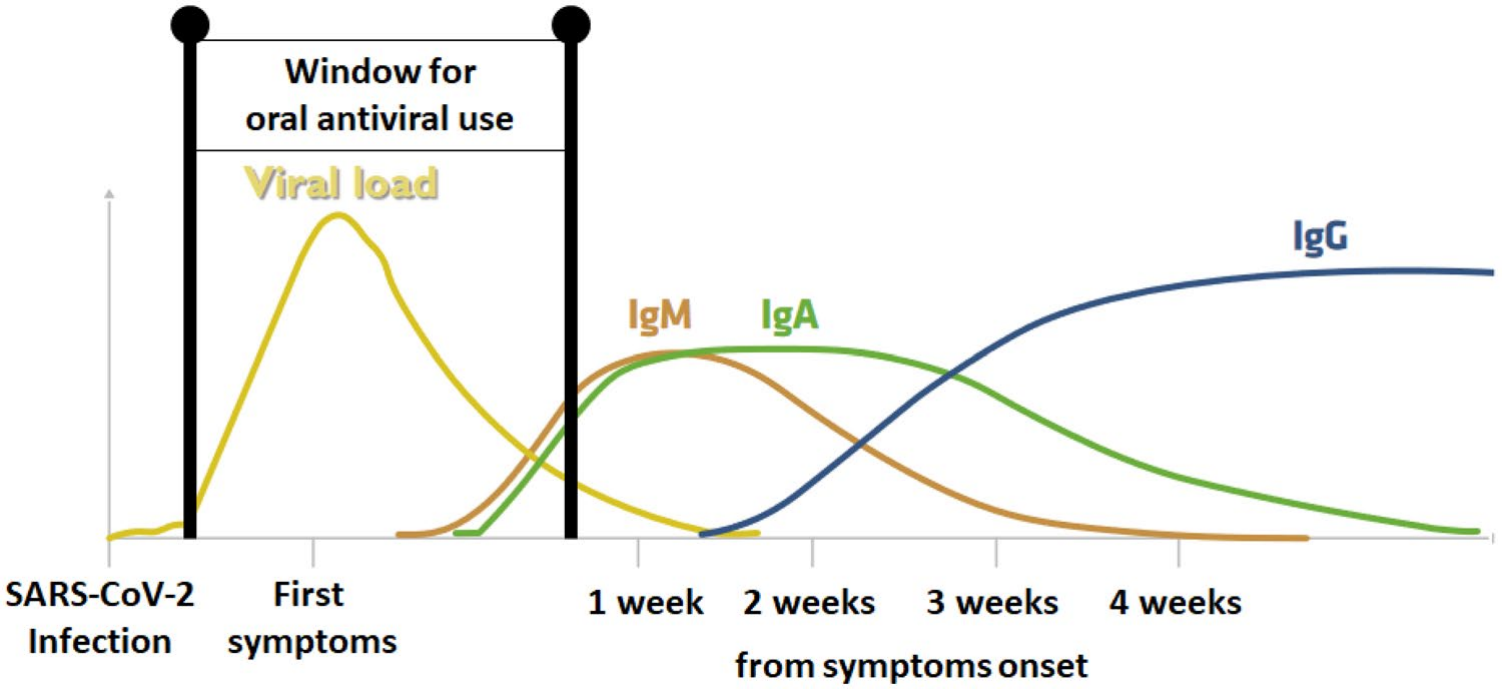
The typical viral load dynamics in SARS-CoV-2 infection in immunocompetent patients, humoral response to the infection, and optimal timing for oral antiviral use in COVID-19.

All in all, SARS-CoV-2 is unlikely to be eradicated in the near future, while the vaccination strategy has its obstacles [25, 50]. Therefore, concomitant approaches are required to decrease the risk of severe COVID-19 and limit this disease's burden on the healthcare system. This can be achieved, *inter alia*, by developing oral antiviral pharmaceuticals that could be applied outside the clinical setting soon after detecting SARS-CoV-2 infection/symptoms (Fig. 1).

However, such development is a time-consuming process, which requires a good understanding of viral biology, selection of appropriate viral-based or host-based targets, designing the novel chemical compound that can interfere with these targets in the desired manner (e.g., inhibition), and then testing the candidate in vitro and in vivo in the preclinical phase and eventually, in large randomized, placebo-controlled trials. Nevertheless, oral antivirals for COVID-19 treatment may represent a milestone in controlling SARS-CoV-2 and decreasing the progression of the disease into more severe form, particularly in high-risk individuals, including the elderly and individuals with obesity and comorbidities such as cancer, cardiovascular disease, diabetes, and immunosuppression [51–53]. Therefore, their development, testing, and authorization should be viewed as a companion strategy in COVID-19 management, not as a competitor to vaccination.

This review paper aims to identify and characterize the potential host-based and viral-based targets for oral antivirals development to treat COVID-19, highlight and compare the main features, advantages, and limitations of the first-generation oral antiviral therapeutics (nirmatrelvir/ritonavir and molnupiravir), and present the potential future generations of this class of pharmaceuticals currently in different phases of the clinical trials.

## Pharmaceutical targets of SARS-CoV-2

### Host-based pharmaceutical targets

#### At the level of angiotensin-converting enzyme 2

The primary receptor facilitating SARS-CoV-2 entry to the cell is angiotensin-converting enzyme 2 (ACE2) [54]. Among various cell types and tissues, the ACE2 receptor is also present in the mouth and nasal epithelial cells, while its increased expression in the lungs is correlated with age. This, at least partially, explains the higher viral load and severity of symptoms observed in elderly individuals who contracted SARS-CoV-2 [55]. ACE2 was already known for mediating infection of SARS-CoV-1 by recognizing the receptor-binding domain of the spike protein (S1-RBD) with the α-helical region located in the peptidase domain [56, 57]. Therefore, inhibition of ACE2 by drug target represents a potential for treating SARS-CoV-2 infection [58]. Anti-SARS-CoV-2 therapies at the level of ACE-2 may, for example, be based on interfering with the virus − host ACE2 − S1-RBD interface. This would require using low-weight molecules with the ability to negatively interact with the efficiency in the dynamic network of protein–protein interaction that is pivotal for viral entry to the cell [59, 60]. To this end, numerous drug candidates have been identified from the Food and Drug Administration database through an in silico-guided repurposing study. However, they have not been shown to decrease the virus infection [60].

Furthermore, therapies based on ACE inhibitors or angiotensin blockers may be used for organ-protection purposes, e.g., myocardial injuries. For this purpose, telmisartan has been suggested for the potential treatment of COVID-19, but the real impact of ACE inhibitors on SARS-CoV-2 remains controversial [59]. As SARS-CoV-2 down-regulates the expression of ACE2, the delivery of soluble ACE2 may potentially have a therapeutical effect by competing with the host ACE2 for binding with spike protein. To this end, soluble recombinant ACE2/APN01, which mimics the human ACE2, has been proposed to bind SAR-CoV-2, leading to the inactivation of the virus through its inability to interact with ACE2 on the cell surface [60]. An example of such a molecule is GSK2586881, a recombinant human ACE2, which safety and tolerability were investigated before the COVID-19 pandemic in phase IIa clinical trial (NCT01597635) that involved patients suffering from acute lung injury or acute respiratory distress syndrome. However, to the best of our knowledge, by May 2022, no such molecule has entered clinical trials as a candidate for COVID-19 treatment. However, one should note that some patients with COVID-19 tend to have an increased concentration of soluble ACE2, yet they remain prone to severe disease. As suggested, the complex of SARS-CoV-2 with soluble ACE2 version may potentially lead to the viral spread to distant organs and other events that may worsen the clinical course of the disease [61]. Moreover, soluble ACE2 has also been shown to facilitate infection of SARS-CoV-2. [62, 63] These aspects must be carefully addressed for any soluble ACE2 candidates for COVID-19 treatment.

#### At the level of transmembrane serine protease 2 activation

Following the interaction of SARS-CoV-2 with a receptor, various host proteases activate the virus − host cell membrane fusion for subsequent delivery of the viral genome. The cell surfaces of the respiratory tract of the human host and those of the gastrointestinal and urogenital systems express transmembrane serine protease 2 (TMPRSS2), which was already known to activate spike protein of SARS-CoV and MERS-CoV [57, 64]. TMPRSS2 cleavage of SARS-CoV-2 spike protein is the favored route for viral infection over other proteases, including the endosomal cathepsins [65]. Hence, TMPRSS2 inhibitors have been proposed as a potential drug target for COVID-19 therapy. Other drugs with protease inhibitory actions include 4-(2-aminoethyl) benzenesulfonyl fluoride and nafamostat [66, 67]. Repurposing the mucolytic drug bromhexine and transcriptional inhibition of TMPRSS2 may also offer some therapeutic options for COVID-19 [60]. During the COVID-19 pandemic, a novel human extracellular inhibitor of TMPRSS2, i.e., alpha 1 antitrypsin, was discovered. This has lead to the suggestion that treatment with this or similar extracellular protease inhibitor, may be beneficial for patients with COVID-19 [68].

As recently shown, a small-molecule compound, N-0385, that inhibits TMPRSS2, successfully blocked the entry of different SARS-CoV-2 variants in vitro, with its potency also evidenced in vivo [69]. This indicates that it may be an effective treatment option if applied early during SARS-CoV-2 infection. Importantly, TMPRSS2 inhibitors may not be effective in patients who are already hospitalized with COVID-19, as shown in a double-blind, randomized, placebo-controlled trial of camostat mesylate, which failed to reduce time to clinical improvement, clinical progression of the disease, or risk of death [70].

#### At the level of furin cleavage

Furin is a type 1 membrane-bound protease that belongs to the subtilisin-like proprotein convertase family with high levels of expression in the lungs [60]. The spike protein of SARS-CoV-2 expresses a highly cleavable furin-like site that increases the pathogenicity of the virus over other β-coronaviruses [71]. It is established that the insertion of such cleavage sites in other coronaviruses, e.g., in the infectious bronchitis virus, enhanced the pathogenicity, leading to neural symptoms in chickens [60]. Furthermore, mutation near the furin cleavage site (F1-2) affects the spike protein's electrostatic distribution and surface structure and impairs its ability to bind to furin. This confirms that interaction between furin and ACE2 plays an important role in the infection of cells by SARS-CoV-2 [72]. Therefore, inhibiting furin with peptides and, more recently, with small molecules may represent a potential therapeutic option to halt inflammation and viral infection [73]. Several compounds with potency inhibit human furin, e.g., decanoyl-Arg-Val-Lys-Arg-chloromethylketone, α1-antitrypsin Portland or succinoyl esters of andrographolide isolated from the herbaceous plant *Andrographis paniculate* (Burm.f.) Nees [74]. As evidenced, furin inhibitor decanoyl-RVKR-CMK has been demonstrated to inhibit the processing of SARS-CoV-2 successfully. However, according to our knowledge, no molecule revealing such a mechanism of action has so far entered a clinical trial for the treatment of COVID-19.

#### At the level of cathepsin L activation

Cathepsin L activation of SARS-CoV-2 spike protein by cleavage of proteases is one of the crucial steps in viral infection [75]. Several lysosomal cathepsins are facilitating the entry of human coronaviruses by endocytosis. However, in the case of SARS-CoV-2, only cathepsin L plays an important role in this regard [76]. Some studies reported that selective inhibitors of cathepsin L could reduce the entry of SARS-CoV-2 pseudovirus into HEK 293/hACE2 cells suggesting a plausible role of cathepsin L in priming of spike protein in the lysosome [54]. Furthermore, a synergistic effect may also be achieved by simultaneously targeting TMPRSS2 and cathepsin L. However, the selectivity of this strategy remains a challenge and must rely on several computational methods using data derived from the established 3D structures [77].

Therefore, cathepsin L inhibitors have been proposed as therapeutic options for COVID-19 [60, 75]. Some molecules exhibiting such a mechanism of action have been developed, e.g., MPI8, a simultaneous inhibitor of SARS-CoV-2 main protease [78]. However, preclinical and clinical evidence is required to assess whether cathepsin L inhibitors may show a benefit in COVID-19 treatment.

#### Adaptor-associated kinase 1 (AAK1) and cyclin G-associated kinase (GAK) inhibition

Adaptor-associated kinase 1 (AAK1) and cyclin G-associated kinase (GAK) are serine-threonine protein kinases. It is established that they can control intracellular viral trafficking as well as viral assembly and release of virions of various unrelated RNA viruses (e.g., dengue, Ebola, hepatitis C virus, or rabies) [79]. AAK1 plays an essential function in receptor-mediated endocytosis by specific phosphorylation of adaptor protein 2, which stimulates the binding to cargo proteins, whereas GAK controls clathrin-mediated trafficking and facilities binding of clathrin to the trans-Golgi network and plasma membrane [80]. Because the main pathway of SARS-CoV-2 entry is receptor-mediated endocytosis, it is proposed that the regulatory effects of these protein kinases could be optimized to reduce viral entry [60]. For this purpose, several molecules with antiviral effects have been tested for their capacity to inhibit AAK1 activity. For example, baricitinib (a potent AAK1 and GAK inhibitor) has been suggested as an effective treatment option in COVID-19 because of its ability to impair viral entry into host tissues [81]. The clinical trials have shown that oral administration of baricitinib in addition to standard of care (including dexamethasone) had a similar safety profile to that of a standard of care alone in hospitalized adults with COVID-19 but was related to reduced mortality [82]. Food and Drug Administration has authorized baricitinib for emergency use along with remdesivir. It can be used in adults and children > 2 years who require oxygen supplementation, invasive mechanical ventilation or extracorporeal membrane oxygenation). The recommended dosage is 2 mg for individuals < 9 years and 4 mg for older patients taken daily for 14 days [83].

#### Phosphatidylinositol 3-Phosphate 5-Kinase (PIKfyve) inhibition

The enzyme phosphatidylinositol 3-phosphate 5-kinase (PIKfyve) is a potential drug target for the treatment of infections caused by viruses that enter host cells through endocytosis, including SARS-CoV-2 [60, 84]. PIKfyve has a lipid kinase activity but also serves as a protein kinase that mediates the synthesis of phosphatidylinositol-3,5-bisphosphate. It is one of the essential molecules that regulate the endosome maturation during endocytosis as well as trafficking events related to the endocytic pathway [85]. Recently, Ou et al. [76] confirmed that the cellular entry of SARS-CoV-2 was reduced after treatment with apilimod, a potent inhibitor of PIKfyve, which also inhibits the production of interleukins-12 and -23 [86, 87]. Moreover, in vitro studies indicate that XMU-MP-7, a novel PIKfyve inhibitor, plays antiviral roles in the entry and post-entry phase and effectively blocks viral infections of different SARS-CoV-2 variants (including Delta and Omicron variants) [88]. However, the use of PIKfyve inhibitors in COVID-19 treatment requires clinical evidence.

### Virus-based pharmaceutical targeted sites

#### At the level of the RNA-dependent RNA polymerase (RdRp) activation

RNA-dependent RNA polymerase (RdRp), also known as nsp12, is a highly conserved protein in SARS-CoV-2, critical for RNA transcription and viral replication. The RdRp domain of the polymerase is situated at the C-terminus and has a conserved Ser-Asp-Asp motif. It is known that the enzymatic activity and binding of nsp12 to RNA are increased by the nsp7–nsp8 complex [89]. The active site of nsp12 binds the RNA genome for the synthesis of new strands, and daughter genomes are subsequently produced through the replication process [90]. Therefore, blocking this binding can inhibit viral replication and is a promising target for drug development [90]. There are two ways of inhibiting the replication of the virus. First is based on the insertion of non-canonical RNA or DNA nucleotide into the RNA—this nucleotide is not recognized by RdRp during the replication, and consequently, the chain-elongation reaction of the viral RNA should be stalled. Remdesivir, a non-canonical nucleotide, inhibits the replication of the SARS-CoV-2 via this pathway [91]. In a second pathway, an inhibitor binds to RdRp to block the entry of the viral RNA into the active site of RdRp for further processing [90]. Because of its membrane-permeable backbone, remdesivir can easily reach the cytoplasm, where it is converted to remdesivir monophosphate and to its final form of the remdesivir triphosphate, which covalently binds to the viral RNA and inhibits the replication [90]. Based on clinical evidence, the use of remdesivir has been authorized to treat COVID-19 in adults and adolescents (> 12 years with weight ≥ 40 kg) who require oxygen therapy. It can also be used in adults who do not require oxygen supplementation but who represent high-risk group for severe COVID-19 [92].

Similar to remdesivir, the triphosphate form of the pyrimidine-based antiviral drug, favipiravir-ribofuranosyl-5′-triphosphate (favipiravir-TP) was also evidenced to bind the RdRp [93, 94]. Favipiravir-TP is either incorporated into the viral RNA strand and inactivates the RNA-chain-elongation reaction or binds to the conserved residues of the RdRp, thereby inhibiting the entry and exit of the viral RNA [90]. The use of favipiravir, also in oral form, is subject to clinical trials (see Sect. 4) with contradictory results, possibly due to a different time of its administration in COVID-19 patients [95]. Triazavirin, a guanine nucleotide analog, directly targets the RNA-chain termination reaction to halt SARS-CoV-2 replication [96]. However, its binding to RdRp is weaker than remdesivir [73], and the clinical evidence does not support that triazavirin could significantly benefit the COVID-19 patients [96]. Similarly, ribavirin, a guanosine analog, interferes with RNA replication by inhibiting the RdRp protein's activities of the SARS-CoV-2 [97]. However, ribavirin also inhibits the formation of endogenous guanosine by directly inhibiting inosine monophosphate dehydrogenase [98]. Observational studies indicated that it might increase the SARS-CoV-2 clearance in critically ill patients [99]. For this reason, ribavirin entered clinical trials to investigate its potential use in COVID-19 treatment [90]. Moreover, ribavirin was also evidenced in vitro to downregulate the expression of ACE2 and TMPRSS2, which may additionally decrease the ability of SARS-CoV-2 to infect cells and propagate [100]

Galidesivir, an adenosine nucleoside analog, has also been proposed to block the RdRp of SARS-CoV-2 because of its potency against the RNA polymerase of the Ebola, Yellow Fever, and Zika viruses [90, 97]. Due to promising in vivo data, galidesivir has entered clinical trials for potential treatment in COVID-19 [101]. Other drugs like analogs of dabigatran etexilate 6ʹ-fluorinated-aristeromycin, penciclovir, novobiocin, chenodeoxycholic acid, idarubicin, acyclovir, and fleximer analogs also revealed the potency to inhibit the RdRp [89]. Interestingly, some natural anti-inflammatory compounds with antiviral effects, i.e., gnidicin and gniditrin synthesized by *Gnidia lamprantha* Gilg and betulonal obtained from *Cassine xylocarpa* Vent., have a high binding affinity to RdRp, indicating that they might be a promising avenue to explore in COVID-19 treatment [102]. However, further in vivo and clinical assessments are required.

#### At the level of papain-like protease and main protease

In all coronaviruses, papain-like protease (PL^pro^) and main protease (M^pro^, formally known as 3-chymotrypsin-like protease or 3CL^pro^) are important for the release of several non-structural proteins (nsp) from the amino-terminal end of polyproteins 1a and 1ab [103]. The M^pro^ cleaves off nsp4-nsp16, while PL^pro^ releases nsp1-nsp3 [90]. Both proteases are essential for the replication and translation of SARS-CoV-2 [58]. Furthermore, PL^pro^ of SARS-CoV has de-ubiquitinating and interferon antagonism effects and subsequently prevents activation of interferon-regulatory factor 3 and inhibits the nuclear factor-j-light-chain-enhancer of activated B cells pathway [104]. Therefore, the inhibition of these two proteases is expected to suppress replication and translation of the SARS-CoV-2 and was suggested as a promising antiviral drug target [105, 106]. For instance, using a combination of lopinavir and ritonavir can decrease the virus load in COVID-19 patients [107]. It is also active against SARS-CoV-1 and MERS-CoV and [108]. Some structural studies demonstrated that lopinavir/ritonavir could bind to the active site of M^pro^ of SARS-CoV-2 [109]. Due to this, lopinavir/ritonavir was initially recommended for COVID-19 treatment, but its efficacy was not confirmed in clinical trials—therefore, the use was eventually abandoned [110]. M^pro^, also known as nsp5, mediates nsps maturation required for the virus's life cycle. First, it is cleaved from polyproteins and produces mature enzymes. Second, cleaves downstream nsps at 11 sites leading to the release of nsp4–nsp16 [111]. Clinically valuable drugs, including conivaptan, ledipasvir, montelukast, nicardipine, telmisartan, and velpatasvir, revealed the most significant binding affinity to M^pro^ than peptide inhibitors and small-molecule inhibitors [89, 112–114]. Moreover, computational screening studies have also recognized that several antivirals (e.g., ribavirin), antibacterial (e.g., chloramphenicol), and antitussive (e.g., levodropropizine), and muscle relaxant drugs (e.g., chlorphenesin carbamate) have inhibitory effects against PL^pro^ protease activity of the SARS-CoV-2 [102]. However, in vivo evaluations of both potency and toxicities of such inhibitors are required before using them against SARS-CoV-2 [90, 115, 116]. The first SARS-CoV-2 protease inhibitor with clinically evidenced efficacy in preventing a progression to severe disease is nirmatrelvir/ritonavir, which characteristics are discussed in detail in Sect. 3 of this paper.

#### At the level of helicase activity, nsp-1 and nsp-9

NTPase/helicase (nsp13) catalyzes the separation of duplex nucleic acids into single strands in reactions driven by the hydrolysis of adenosine triphosphate and plays a key function in the replication − transcription complex of coronaviruses. NTPase/helicase, a superfamily 1 helicase, is a multi-functional protein with an N-terminal metal-binding domain and a helicase domain. The N-terminal domain forms a Zn-binding domain, while the C-terminal region forms a helicase domain with a conserved motif that participates in re-arranging double-stranded viral DNA and RNA along the 5′–3′ direction in a nucleoside triphosphate-dependent manner. The nsp13-dependent unraveling is an essential process for the replication, transcription, and translation of SARS-CoV-2 and is considered a promising target for anti-COVID-19 pharmaceuticals [117]. Some potent inhibitors of NTPase/helicases encoded by SARS-CoV-2, such as derivatives of oligo-oxa-adamantanes, e.g., bananins and 5-hydroxychromone derivatives are a subject to preclinical studies for the treatment of COVID-19—they inhibit both the ATPase and the helicase activity of the nsp-13 from SARS-CoV and block its replication of the virus [60, 89]. The nsp-1 protein inhibits gene expression in host cells and induces a shutdown of translation of host proteins by binding to the ribosomal subunit. The binding of nsp-1 leads to the endonucleolytic cleavage and subsequent degradation of the host mRNAs resulting in the inhibition of all cellular antiviral mechanisms. Therefore, the inhibition of nsp-1 represents a potential target site for SARS-CoV-2 drugs as it would be beneficial to developing host cell immune response and reducing viral replication [118]. Recently, montelukast, a drug approved for asthma management, has been shown to bind nsp-1 and minimize replication of SARS-CoV-2 in cell cultures [119]. However, further investigations are required to understand whether it may provide any benefit in COVID-19 treatment. Moreover, the nsp-9 of beta-coronaviruses was proposed to bind host RNA via a unique fold α-helical motif close to the dimer interface of the peptide-binding site [90]. It is suggested that the disruption of the dimer interface of nsp-9 by novel therapeutic candidates may block the binding of RNA to nsp-9 and inhibit its function [120]. However, these molecules and their activities are currently in an early stage of development or in vitro studies [121, 122].

#### At the level of the envelope (E), nucleocapsid (N), and membrane (M) proteins

The E-protein plays a significant role in SARS-CoV-2 assembly, budding, envelope formation, membrane permeability, and overall pathogenesis [123]. Structurally, SARS-CoV E-protein has a PDZ-binding motif in the C-terminus that binds to host adapter proteins taking part in the viral infection [124]. It is thought that molecules that can bind the PDZ domain of SARS-CoV E may have a direct role in viral pathogenesis [125]. For instance, PDZ domains in different coronaviruses bind to a host protein, syntenin, resulting in the induced cytokine storm [90]. In the case of SARS- CoV-2, two different domains constitute the E-protein: the charged cytoplasmic tail on the C-terminus and the hydrophobic domain with the N-terminus being translocated across the membrane [89]. Hence, it is reasonable to hypothesize that inhibition of the N-terminal domain can block SARS-CoV-2 fusion, transcription, and replication.

Furthermore, during fusion, the transmembrane region of E-protein creates an ion channel in the host cell membrane altering their transmembrane potential. It is proposed that this serves as a marker of viral infection and can be inhibited to suppress viral activities [126]. Drugs such as hexamethylene amiloride, amantadine, and some phytochemicals have been used to inhibit the ion channel activities of E-protein of different coronaviruses [126–128]. However, there are yet no specific inhibitors of E-protein of SARS-CoV-2 identified. The common domain architecture of coronavirus N protein consists of three highly conserved regions [129]. The central role of the N protein N-terminal domain is to bind the viral RNA genome [129]. This is critical for the generation of highly ordered viral RNA conformation suitable for transcription and replication. The C-terminal domain is responsible for dimerization, while the conserved Ser/Arg-rich linker regions are responsible for phosphorylation [90]. Recent findings on the crystal structure of the N-terminal domain of SARS-CoV-2 have identified a nucleotide-binding site for the viral RNA, which nucleotide drugs can inhibit to suppress the activities of the N protein [130].

On the other hand, the M-protein is the most abundant coronaviral protein that plays an essential role in the assembly of virions [125]. The most significant function of the M-protein is to regulate the incorporation of the S-proteins into the viral envelope. During this event, the M-protein also interacts with other coronavirus proteins, including the N and S-proteins, allowing the assembly of new virions [125]. However, the M–M interactions form the bulk of the scaffold for the viral envelope [131]. Therefore, chemical compounds that can disrupt the M-S and M–N protein interactions are suggested to inhibit the assembly and packaging of SARS-CoV-2. Overall the E, N, and M proteins are important pharmaceutical targets for developing antiviral medicines for COVID-19 treatment. Interestingly, small interfering RNAs (siRNAs) and several clinically valuable drugs found strong binding to these targets, inhibiting viral replication in the host cells [102]. However, clinical evidence for using these molecules in COVID-19 is still lacking.

## First-generation oral antivirals for COVID-19 treatment

The advent of COVID-19 vaccines and antivirals has significantly reduced the mortality, hospitalizations, and incidence of SARS-CoV-2 infection [25, 35, 132, 133]. The arrival of COVID-19 oral antivirals marked another significant step toward treatment accessibility, convenience, and time-effectiveness, which would lead to an unprecedented opportunity to improve COVID-19 outcomes. One should note that oral antivirals are the parallel strategy of decreasing the overall burden of COVID-19, not an alternative to vaccines, which aim to prevent infection and, foremost, severe disease.

**Fig. 2:**
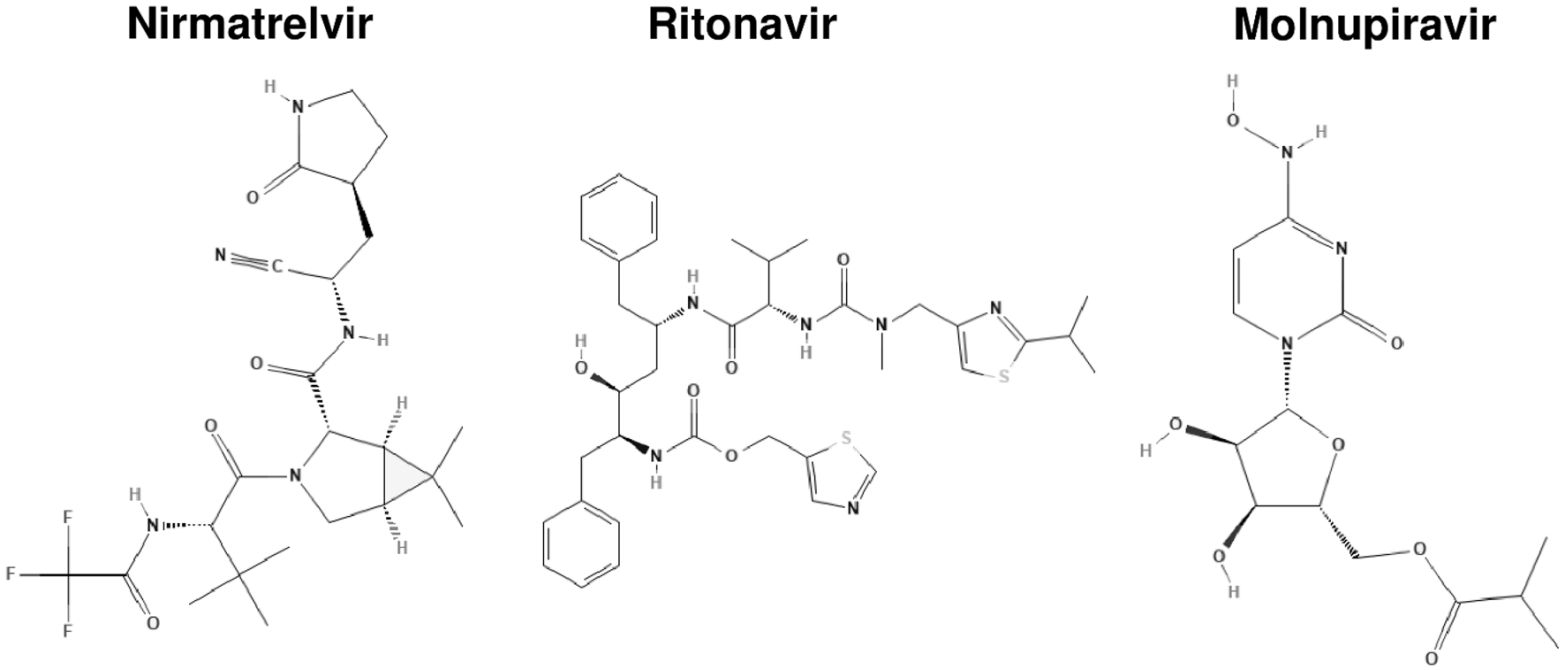
The chemical structures of nirmatrelvir and ritonavir (components of paxlovid), and molnupiravir

**Fig. 3:**
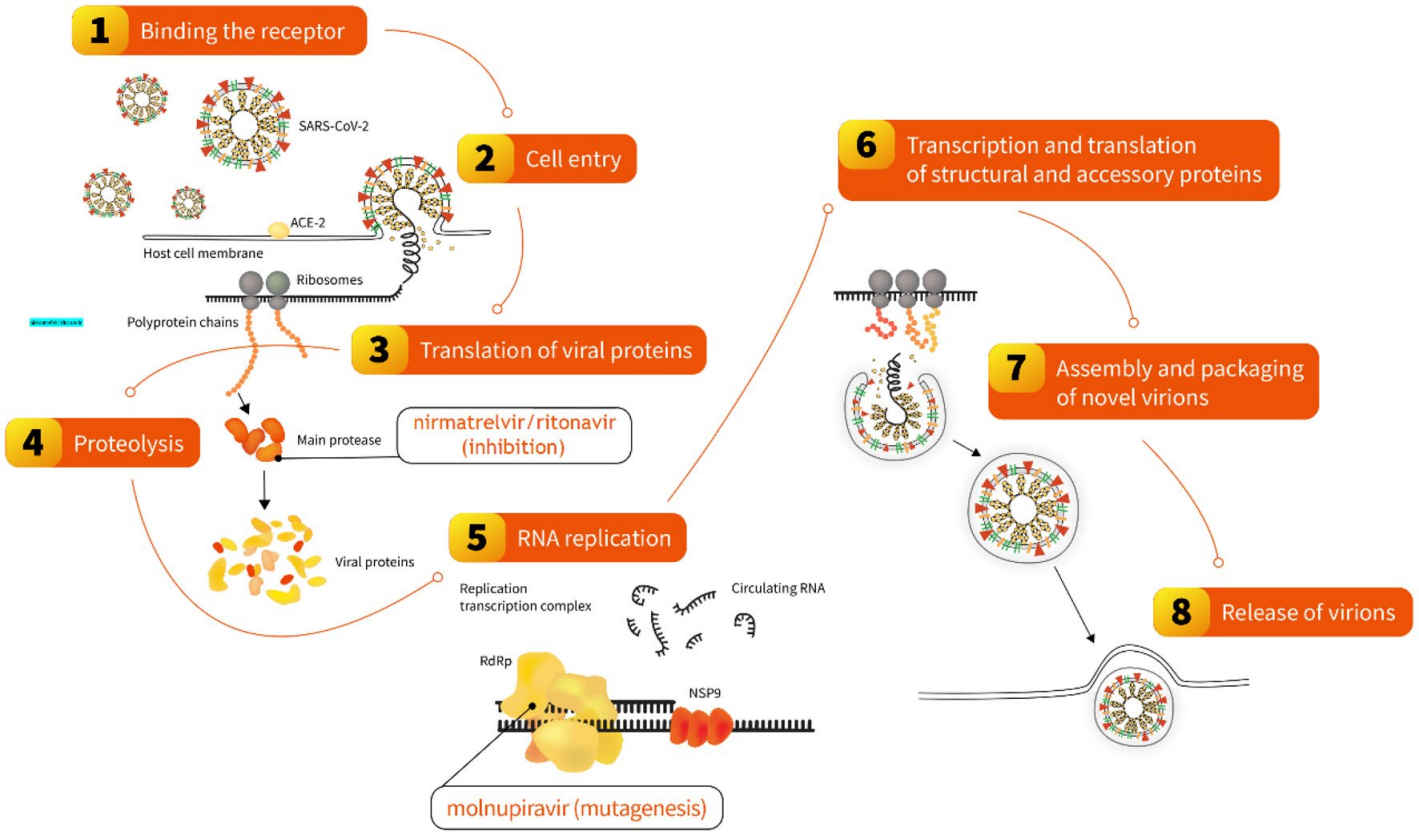
A scheme of SARS-CoV-2 replication in the human cell with steps during which nirmatrelvir/ritonavir and molnupiravir are interfering, ultimately exhibiting antiviral action

Ritonavir-boosted nirmatrelvir (paxlovid) and molnupiravir (lagevrio) are the first two oral antiviral treatments that are authorized to treat mild-to-moderate COVID-19 [134]. Although they differ from each other chemically (Fig. 2) and in terms of mechanism of action (Table 1) and act during two different steps of SARS-CoV-2 replication (Fig. 3), both are intended to be used in patients with early SARS-CoV-2 infection who are characterized by a high risk of developing severe illness and have raised the hopes for better management of SARS-CoV-2 infections [135]. Clinical studies suggest that nirmatrelvir/ritonavir can lower the risk of severe COVID-19, defined as hospitalization or death for high-risk individuals, by almost 90%, and molnupiravir can lower this risk by about 30% (with 89% reduction of death risk) [135]. The recent real-world data confirm the efficacy of both oral antiviral in the large cohort of COVID-19 inpatients. Their use was associated with a shorter time of SARS-CoV-2 elimination and a significantly lower risk of disease progression. Although molnupiravir also had a lower risk of invasive mechanical ventilation, a direct comparison of both antivirals revealed that nirmatrelvir/ritonavir was superior in decreasing the length of hospital stay and mortality [136].

**Table 1:** Comparison of nirmatrelvir/ritonavir and molnupiravir oral antivirals for COVID-19 treatment [135, 137, 138]

	Nirmatrelvir/ritonavir (paxlovid)	Molnupiravir (lagevrio)
Manufacturer	Pfizer	Merck & Co
Drug class	SARS-CoV-2 main protease (M^pro^) inhibitor (nirmatrelvir); HIV-1 protease inhibitor & CYP3A inhibitor (ritonavir)	Nucleoside analog
Chemical formula	C_23_H_32_F_3_N_5_O_4_ (nirmatrelvir) C_37_H_48_N_6_O_5_S_2_ (ritonavir)	C_13_H_19_N_3_O_7_
Mechanism of Action	Inhibits M^pro^, preventing viral replication	Viral lethal mutagenesis
Dosing	300 mg nirmatrelvir with 100 mg ritonavir every 12 h *	800 mg every 12 h
Pills per dose	3	4
Treatment initiation	Within 5 days from symptom onset	Within 5 days from symptom onset
Treatment duration	5 days	5 days
Total dose	4000 mg (3000 mg nirmatrelvir + 1000 mg ritonavir)	8000 mg
Efficacy against hospitalization/death	89% [139]	30% [140] (89% risk of death reduction)
Indication	At-risk patients with mild-moderate COVID-19	At-risk patients with mild-moderate COVID-19
Age limit	12 years or older	18 years or older
Renal dose adjustment	For eGFR 30 to 60 mL/min/1.73 m^2^ Avoid if eGFR below 30 mL/min/1.73 m^2^	None
Hepatic dose adjustment	Avoid in severe hepatic impairment (Child–Pugh Class C)	None
Contraindications	Hypersensitivity to ingredients Use with certain drugs that have CYP3A4 interactions	None listed
Special populations	No human data on use in pregnancy or breastfeeding	Not recommended in pregnancy Not recommended if breastfeeding
Most common adverse reactions	Dysgeusia, diarrhea, hypertension, myalgia	Diarrhea, nausea, dizziness
Warnings	Various drug interactions Hepatotoxicity HIV-1 drug resistance in patients with HIV-1 infection	Embryo-fetal toxicity Bone and cartilage toxicity

^*^—there might be a need to adjust the dose for some specific groups of patients, e.g., with chronic disease – for details, see [141]

### Nirmatrelvir/ritonavir (paxlovid)

#### Main characteristics

Nirmatrelvir was demonstrated to reveal antiviral activity against all coronaviruses that are known to infect humans [142–146]. Paxlovid (nirmatrelvir/ritonavir) contains nirmatrelvir, with addition of ritonavir, a potent cytochrome P450 (CYP) 3A4 inhibitor and pharmacokinetic boosting agent that has been used to boost HIV protease inhibitors [142, 144]. Co-administration of ritonavir is required to increase concentrations of nirmatrelvir to the therapeutically active range [142, 144].

Nirmatrelvir/ritonavir is an investigational medicine that was authorized on 22 December 2021 by the Food and Drug Administration for emergency use to treat mild-to-moderate COVID-19 in adults and children aged ≥ 12 years and older weighing at least 40 kg with positive results of direct SARS-CoV-2 viral testing, and who are at high risk for progression to severe COVID-19, including hospitalization or death [143, 144, 147]. Its efficacy is reported to continue in the presence of the Omicron variant [148]. Nirmatrelvir/ritonavir can only be obtained with a prescription [143, 144]. The recommended dose is two tablets, each containing 150 mg nirmatrelvir, plus one tablet containing 100 mg ritonavir, to be administrated orally twice a day for 5 days in non-hospitalized patients [143, 144, 147]. Treatment with nirmatrelvir/ritonavir should be initiated immediately after a SARS-CoV-2 infection has been diagnosed, within 5 days of the onset of first COVID-19 symptoms. The tablets should be stored at room temperature (20–25 °C) [144].

#### Mechanism of action

The mechanism of nirmatrelvir action is to inhibit M^pro^, which disables its ability to process polyprotein precursors (Fig. 3), ultimately leading to the inability of the virus to replicate [143, 146]. A low dose of ritonavir is required as it inhibits the CYP3A-mediated metabolism of nirmatrelvir, slowing its breakdown and increasing the durability of its circulation at levels that affect the multiplication of the virus. Nirmatrelvir/ritonavir aims to eliminate the viral infection at the early symptomatic phase and prevent the progression to a more severe clinical form [143, 149].

#### Side effects

The use of nirmatrelvir/ritonavir may result in some side effects (which may influence less than one in ten individuals), of which the most common include headache, diarrhea, taste disturbance (dysgeusia), and emesis [139, 143, 150]. Nirmatrelvir/ritonavir can possibly cause allergic reactions as its side effect after only taking one dose [144]. Following are symptoms of an allergic reaction by nirmatrelvir/ritonavir, such as hives; trouble in swallowing or breathing; swelling of the mouth, lips, or face; throat tightness; hoarseness; and skin rash [150]. Liver function can also be affected by nirmatrelvir/ritonavir with symptoms such as appetite loss, yellowing of the skin, and the whites of eyes (jaundice); dark-colored urine; pale-colored stools and itchy skin; and stomach area (abdominal) pain. Nirmatrelvir/ritonavir can also cause resistance to HIV medicines. Other possible side effects include diarrhea, altered sense of taste, high blood pressure, and muscle aches. These are not all the possible side effects of nirmatrelvir/ritonavir—the drug is still under various investigations, so it is plausible that all of the potential risks are currently unknown [144].

#### Interactions

Nirmatrelvir/ritonavir has significant and complex drug–drug interactions, mainly due to the presence of ritonavir [142, 144]. Before prescribing nirmatrelvir/ritonavir, clinicians must carefully review the patient's concomitant medications, including over-the-counter medications, herbal supplements, and recreational drugs [151]. Concomitant use of nirmatrelvir/ritonavir with numerous drugs may impact its efficacy or cause serious and life-threatening side effects; these drugs include alfuzosin, pethidine, propoxyphene, ranolazine, amiodarone, dronedarone, flecainide, propafenone, quinidine, colchicine, lurasidone, pimozide, clozapine, dihydroergotamine, ergotamine, methylergonovine, lovastatin, simvastatin, sildenafil for pulmonary arterial hypertension, triazolam, oral midazolam, apalutamide, carbamazepine, phenobarbital, phenytoin, rifampin, and formulas based on St John's wart (*Hypericum perforatum* L.) [147]. Post-authorization surveillance of potential drug interactions is recommended.

#### Clinical data of nirmatrelvir/ritonavir

The main, ongoing or initiated clinical trials on nirmatrelvir/ritonavir are summarized in Table 2. The pivotal phase 2–3 double-blind, randomized, controlled trial on nirmatrelvir/ritonavir conducted in symptomatic, unvaccinated, non-hospitalized adults at high risk for progression to severe COVID-19 reported a reduction of hospital admission or death by 97% relative to placebo [139]. Patients received nirmatrelvir/ritonavir treatment for five days since symptoms onset and outcome, viral load, and safety were monitored for 28 days. Over the month following treatment, the rate of hospitalization or death was 0.8% (8 out of 1,039) for patients who received nirmatrelvir/ritonavir, compared with 6.3% (66 out of 1,046) for those who received placebo. There were no deaths in the group receiving nirmatrelvir/ritonavir, while 13 deaths occurred in the placebo group. Most patients in the study were infected with the Delta variant [139]. However, laboratory studies have shown that nirmatrelvir/ritonavir is also active against Omicron and other SARS-CoV-2 variants [152, 153]. Moreover, the recent real-world retrospective study involving a large inpatient cohort during Omicron BA.2 variant domination confirmed the high efficacy of nirmatrelvir/ritonavir in shortening viral replication, reducing disease progression, and preventing death [136].

**Table 2:** List of main ongoing and initiated clinical trials of nirmatrelvir/ritonavir by the end of May 2022

ClinicalTrials.gov identifier	Recruitment status (May 2022)	Study Comments and results
NCT04960202	Active, not recruiting	Phase 3 double-blind, placebo-controlled trial of nirmatrelvir/ritonavir in non-hospitalized high-risk adults with symptomatic SARS-CoV-2 infection. The risk of progression to severe disease was reduced by 89% in participants using nirmatrelvir/ritonavir with no evident safety issues [139]
NCT05386472	Not yet recruiting	No results yet. Phase 1 open-label trial evaluating the pharmacokinetics, safety and tolerability of nirmatrelvir and ritonavir in pregnant women with mild-to-moderate COVID-19
NCT05366192	Not yet recruiting	No results yet. Phase 4 open-label prospective, two-arms study investigating the safety of different dosing of nirmatrelvir/ritonavir in hemodialysis patients with COVID-19
NCT05261139	Recruiting	No results yet. Phase 2/3 open-label trial evaluating safety, pharmacokinetics, and efficacy of nirmatrelvir/ritonavir in non-hospitalized, symptomatic pediatric participants with COVID-19 who are at risk of progression to severe disease
NCT05263908	Recruiting	No results yet. A post-marketing study examining the safety and effectiveness of nirmatrelvir/ritonavir under actual medical practice
NCT05341609	Recruiting	No results yet. Phase 3 study evaluating the safety and efficacy of oral nucleoside analog VV116 (JT001) against SARS-CoV-2, in which nirmatrelvir/ritonavir is used as a comparator oral drug
NCT04381936	Recruiting	No results yet. Phase 2/3 RECOVERY trial for evaluation of 18 different COVID-19 treatment options, including nirmatrelvir/ritonavir
NCT05321394	Recruiting	No results yet. Phase 3 open-label trial on the use of nirmatrelvir/ritonavir or monoclonal antibodies (sotrovimabor, cixagevimab/cilgavimab) in outpatients with mild-to-moderate COVID-19
NCT05263921	Not yet recruiting	No results yet. Phase 1 open-label trial of bioavailability of single dose of nirmatrelvir/ritonavir in three different delivery vehicles compared to the commercial tablets containing nirmatrelvir/ritonavir in healthy adults under fasted conditions

### Molnupiravir

#### Main characteristics

Molnupiravir (lagevrio) was the first oral antiviral drug in the history of COVID-19 treatment, with the UK becoming the first to authorize its use (conditional marketing authorization) on 4 November 2021 [140, 154]. This decision was criticized by some researchers as immature and based on the interim data underlying the press release of the manufacturer [155]. On 23 December 2021, Food and Drug Administration issued an Emergency Use Authorization for molnupiravir for the treatment of adults with mild-to-moderate COVID-19 who are within 5 days of symptom onset, who are at high risk of progressing to severe disease, and for whom alternative antiviral therapies are not accessible or clinically appropriate [156]. In response to growing interest in molnupiravir in European Union, European Medicines Agency has issued an interim recommendation to support national authorities in case they decide on the use of molnupiravir before marketing authorization [157]. The evaluation of an application for marketing authorization of molnupiravir was initiated by European Medicines Agency at the end of November 2021 [158].

#### Mechanism of action

Molnupiravir is a small-molecule ribonucleoside pro-drug of N-hydroxycytidine (NHC), which can treat RNA viral infections, including SARS-CoV-2 [159]. Prior to the COVID-19 pandemic, it was tested experimentally for potential use against MERS-CoV and SARS-CoV-1 [160, 161]. NHC circulates systemically and is phosphorylated intracellularly to NHC triphosphate. In the next step, NHC triphosphate is incorporated into viral RNA by viral RNA-dependent RNA polymerase (Fig. 3). Subsequently, it misdirects it to induce guanosine-to-adenosine and cytidine-to-uridine transition mutations during viral replication [162, 163]. Ultimately, this leads to an accumulation of deleterious errors (so-called "lethal mutagenesis") throughout the viral genome that eventually aims to render the virus noninfectious and unable to replicate [164, 165].

#### Side effects and issues of concern

The most prevalent adverse effects of molnupiravir are diarrhea, dizziness, and nausea [137]. In the clinical trial of 1433 randomized participants, the frequency of adverse events was similar between the treatment and placebo groups [140]. Furthermore, molnupiravir is not authorized for use in children aged < 18 years due to potential effects on bone and cartilage growth [137]. Molnupiravir is known for its potential embryo-fetal toxicity and is contraindicated for use in pregnant individuals and women of child-bearing age not using contraceptive methods right before or during treatment [164]. Another potential issue associated with molnupiravir that has been raised is whether NHC can be mutagenic to host cells, as suggested by some in vitro evidence in which cells underwent prolonged exposure of 32 days [164]. This issue has also been addressed by in vivo Pig-a mutation assay and Big Blue transgenic assay in rats, indicating no increased mutation rate frequency following exposure to molnupiravir [166]. Moreover, in vitro and in vivo micronucleus assays run by the manufacturer did not induce chromosomal damage [166]. Analysis of lung biopsies of golden hamsters infected with SARS-CoV-2 and treated with molnupiravir did not reveal an accumulation of mutations [167]. Although the current evidence suggests that molnupiravir appears not to pose a significant genotoxic risk in clinical use, further investigations are recommended to fully explore the mutagenic potential of molnupiravir to the host [168].

Some researchers hypothesized that the use of molnupiravir may rarely lead to the development of mutations that are not lethal to the SARS-CoV-2 [169]. However, experiments conducted with MERS-CoV showed that the prolonged presence of NHC generates a low level of resistance [160]. Moreover, as shown in a clinical trial, all patients using molnupiravir displayed a significant reduction of viral load during treatment [140]. Importantly, its use in immunocompromised patients was associated with equal viral elimination compared to immunocompetent patients [170]. However, patients must adhere to treatment guidelines and use it for 5 consecutive days in recommended doses to avoid the potential escape of SARS-CoV-2 from lethal mutagenesis.

#### Interactions

As reported outlined by European Medicines Agency, no substantial risks for clinically relevant interactions of molnupiravir (acting as a victim or perpetrator) with other pharmaceuticals have been identified through limited in vivo testing, assuming the recommended dosing of 800 mg every 12 h for 5 days [171]. Post-authorization surveillance of potential drug interactions is recommended.

#### Clinical trials on molnupiravir

Phase 3, a double-blind, randomized, placebo-controlled trial, which results led to the authorization of molnupiravir by various authorities, involved 1433 participants, of which 716 were assigned to receive molnupiravir and 717 to receive placebo. Participants in the treatment group received 800 mg of molnupiravir (four 200 mg tablets) every 12 h for 5 days. The primary efficacy endpoint for this study was the incidence of hospitalization or death at day 29. As shown, the incidence of this composite endpoint was 31% lower in the molnupiravir group, while the risk of death was 89% lower in the molnupiravir group.

The main, ongoing or initiated clinical trials on molnupiravir are summarized in Table 3. The existing data reports that molnupiravir is reducing the viral load in the nasopharynx, with favorable safety and tolerability profiles in COVID-19 patients receiving 5-day therapy. Moreover, data from these studies show a significant reduction in hospitalization or death in adults experiencing mild or moderate COVID-19. Lee et al. recently suggested that the clinical role of oral molnupiravir in the early treatment of patients experiencing asymptomatic or mild COVID-19 and the prevention of SARS-CoV-2 transmission may become more evident in the near future [172]. We doubt their assertion given a whole legion of new antiviral therapies coming up on the market, presenting higher efficacy and safety profile. Importantly, molnupiravir maintains its action against the Omicron variant (as well as other variants) [153, 173]. Recent real-world data originating from a large cohort of inpatients data during Omicron BA.2 dominance confirms that molnupiravir use lowers the risk of disease progression and mechanical ventilation. However, compared to nirmatrelvir/ritonavir, a reported length of hospitalization and mortality risk was higher in molnupiravir users [136]. A recent real-world study conducted in Poland during the dominance of the Omicron variant evidenced that administration of molnupiravir in hospitalized patients within five days from symptoms onset resulted in reduced mortality (compared to a group receiving no antiviral treatment), especially evident in patients aged over 80 years [218]. Although molnupiravir use did not decrease hospitalization time nor the frequency of mechanical ventilation, patients receiving such pharmacotherapy required oxygen supplementation less often [218].

**Table 3:** List of main ongoing and initiated clinical trials of molnupiravir by the end of May 2022

ClinicalTrials.gov Identifier	Recruitment Status (May 2022)	Study Comments and Results
NCT05386589	Not yet recruiting	No results yet. Phase 1 trial of pharmacokinetics of N-hydroxycytidine following a single oral dose of molnupiravir in participants 18 to 75 years with moderate hepatic impairment and healthy matched controls
NCT05386758	Not yet recruiting	No results yet. Phase 1 trial of pharmacokinetics of N-hydroxycytidine following a single oral dose of molnupiravir in participants 18 to 75 years with severe renal impairment and healthy matched controls
NCT04575584	Terminated	Phase 2/3 randomized, placebo-controlled, double-blinded trial terminated for business reasons. Aimed to evaluate the safety, tolerability, and efficacy of molnupiravir compared to placebo
NCT04575597	Completed	Phase 2/3 randomized, placebo-controlled, double-blinded trial. Early treatment with molnupiravir reduced the risk of hospitalization or death in at-risk, unvaccinated adults with COVID-19 [140]
NCT04405570	Completed	Phase 2a randomized, double-blinded, placebo-controlled trial. Molnupiravir was highly effective at reducing nasopharyngeal SARS-CoV-2 infectious virus and viral RNA and has a favorable safety and tolerability profile [140]. Therapeutic treatment of infected animals with molnupiravir twice a day significantly reduced the SARS-CoV-2 load in the upper respiratory tract and completely suppressed the spread to untreated contact animals [174]
NCT04939428	Recruiting	No results yet. Phase 3 trial to determine the efficacy, safety and tolerability of molnupiravir (MK-4482) compared with placebo for the prevention of laboratory-confirmed COVID-19
NCT04405739	Completed	Phase 2a randomized, placebo-controlled, double-blinded trial. Same results as NCT04405570
NCT04381936	Recruiting	No results yet. Phase 2/3 RECOVERY trial for evaluation of 18 different COVID-19 treatment options, including molnupiravir
NCT04746183	Recruiting	No results yet. Seamless Phase 1/2a trial for the rapid evaluation of candidates for COVID-19 treatment
NCT04392219	Completed	Phase 1 randomized, double-blind, placebo-controlled trial evaluating safety, tolerability and pharmacokinetics of molnupiravir. There were no serious adverse events, and there were no clinically significant findings in the clinical laboratory, vital signs, or electrocardiography [175]

## Other oral antiviral treatments of COVID-19 in clinical trials

Viruses, particularly RNA viruses such as HIV, Dengue fever, Ebola, Zika, Middle MERS-CoV, and SARS-CoV-2, lead the current WHO list of ten global health concerns [176]. The production of antiviral medicines necessitates a thorough understanding of viral biology and pathogenicity, particularly its interactions with the host cell. Humans are known to be infected with more than 220 viruses, although the availability of efficient antivirals is still limited [177, 178].

In 1967, Kates and McAuslan were the first to describe the viral enzyme pox virus DNA-dependent RNA polymerase [179], which served as the first mechanistic basis for selective antiviral drugs. The realm of antiviral drugs expanded rapidly in the herpes area with the development of acyclovir [180] and other inhibitors of DNA polymerases. However, the discovery of HIV/AIDS in 1983 provided a tremendous incentive for developing antiviral medications, resulting in many anti-HIV therapies targeting numerous viral enzymes that were developed in a short time [181].

Since all RNA viruses encode an RNA-dependent RNA polymerase (RdRp), developing an efficient antiviral drug can be followed by expanding the successful structure(s) to other viruses beyond the initial clinical target. This is especially true for polymerase inhibitors. Admittedly, nucleoside analog inhibitors like remdesivir, ribavirin, and favipiravir have been shown to have in vitro and/or in vivo efficacy against a variety of RNA virus families, including filoviruses (Ebola virus), HCV, certain viral hemorrhagic fevers, influenza virus, and coronaviruses (SARS-CoV-1, SARS-CoV-2, MERS-CoV) [98, 182–186].

Protease inhibitors, such as lopinavir/ritonavir, are another unique group of licensed antivirals that are effective against SARS-CoV-1, SARS-CoV-2, and MERS-CoV in the in vitro and in vivo animal model studies. They were initially developed to treat HIV and then HCV infections [187]. In addition to antiviral drugs, immunomodulatory drugs such as interferons (IFNs), which are clinically approved for use in HCV and HBV infections, are also being trialed in COVID-19 patients [60, 188]. Furthermore, similar to IFN, hydroxyquinolines modulate immune responses to change infection outcomes in favor of the host by activating the IFN pathway component IRF3 [189].

The COVID-19 pandemic poses an enormous challenge to healthcare, raising an unprecedented need for rapid drug development. However, new drug development incurs high costs exceeding billions of dollars and usually necessitates a long gestation period of 10–15 years [189]. Along this line, drug repurposing, which was previously shown to be good practice for various diseases, has re-emerged in this pandemic, leading to the licensing of dexamethasone and remdesivir for the treatment of severe COVID-19 [190, 191]. However, more effective treatments are needed to target SARS-CoV-2 pathogenicity. The above-mentioned oral antivirals used for the treatment of viral infections due to viruses that share similar pathogenic mechanisms (i.e., viral protein production in the host cell through RdRPs) with SARS-CoV-2, therefore, appear as good drug candidates, and some early phase and small-scale trials [192, 193] have already suggested the efficacy of some of them. Therefore, the purpose of ongoing trials is to test their efficacy and safety in larger populations, accounting for the study limitations of previous trials.

Apart from nirmatrelvir/ritonavir and molnupiravir, some other oral antivirals for the treatment of COVID-19 entered clinical trials (Table 4). Although the first-generation oral antivirals were clinically demonstrated to be effective to a different extent in preventing hospitalization and deaths [139, 140], and this beneficial effect was further confirmed in real-world observations during the Omicron variant wave [136], there is a pressure to develop novel antiviral pharmaceuticals for oral COVID-19 treatment. First, developing and authorizing novel antivirals will likely increase accessibility to these pharmaceuticals in different locations, including low-income countries. Otherwise, the supply of nirmatrelvir/ritonavir and molnupiravir, even if generic versions would be manufactured, might be too short to meet the demand and likely biased toward developed regions, as already seen in the case of COVID-19 vaccines [25, 40, 194].

**Table 4:** General characteristics of ongoing clinical trials on oral SARS-CoV-2 antiviral treatment

Trial code	Phase	Location	Current drug name	Treatment comparator	Mechanism of action	Primary outcome
*NCT* *04,694,612*	3	Nepal	Oral favipiravir	-Placebo -Injectable remdesivir	Inhibition of viral RdRPs [199]	Clinical improvement in mild (within a time frame of 5 days) and moderate (within a time frame of 10 days) cases
*NCT* *04,918,927*	2	Mexico	Oral favipiravir + oral nitazoxanide	Favipiravir + nitazoxanide placebo	Inhibition of viral RdRPs [199]	The difference in the viral load in the upper respiratory tract after 5 days of treatment
*NCT* *04,494,399*	2	China	Oral ribavirine + injectable interferon β-1b	Standard of care	- Inhibition of IMPDH - Inhibition of mRNA capping - Inhibition of viral RdRPs [199] - Downregulation of interferon-stimulated genes - Enhancement of viral mutagenesis [200]	· Clinical symptoms alleviation within a time frame of 7 days
*NCT* *04,402,203*	2b	Bangladesh	Oral favipiravir	standard of care	· Inhibition of viral RdRPs [199]	· Negative RT-PCR for the virus within a time frame of 4–10 days after initiation of treatment
*NCT* *04,466,241*	2b	Côte d’Ivoire	-Oral lopinavir/ritonavir + telmisartan -Oral lopinavir/ritonavir + atorvastatine	lopinavir/ritonavir	Inhibition of viral proteases [201]	Negative RT-PCR for the virus in naspharyngeal swabs and CRP < 27 mg/L at day 11
*NCT* *04,310,228*	2	China	Oral favipiravir + tocilizumab	-favipiravir -tocilizumab	·Inhibition of viral RdRPs [199]	· Clinical cure (viral load of the respiratory specimen negative for two consecutive times, lung image improved, body temperature returned to normal for more than 3 days, clinical manifestations improved) within a time frame of 3 months
*NCT* *05,014,373*	3	Philippines	Oral favipiravir + best supportive care	oral favipiravir + standard care	· Inhibition of viral RdRPs [199]	· Clinical improvement (axillary temperature ≤ 37.4°c, oxygen saturation measured by pulse oxymeter of > 96% without oxygen inhalation, chest imaging with changes showing improvement) within a time frame of 4 to 28 days
*NCT* *04,396,106*	2	USA	AT-527	placebo	- Actions through its active metabolite AT-9010 - Inhibition of viral RdRPs [199] - Competitive inhibition of the natural nucleoside triphosphate for incorporation into the viral RNA - Potential inhibition of NMPylation [202]	· Proportions of subjects with progressive respiratory insufficiency (defined as a ≥ 2-tier increase in respiratory support methods required to maintain satisfactory oxygenation (SpO2 ≥ 93%), using the 6-tier hierarchical scale of respiratory support methods) at day 14
*NCT* *05,047,783*	2	France, Russia, South Africa	Masitinib	placebo	M^pro^ inhibitor	SARS-Cov-2 load at day 10 in patients with symptomatic mild-to-moderate COVID-19
*NCT* *04,600,999*	3	Hungary	Favipiravir	supportive care	Inhibition of viral RdRPs [199]	· Time to improvement in body temperature, in SPO2, in chest imaging, in negative SARS-COV2 (time frame of 9 months)
*NCT* *04,890,626*	3	Spain	Oral emtricitabine + oral tenofovir disoproxil fumarate	-dexamethasone + baricitinib -no treatment	Nucleoside/nucleotide reverse transcriptase inhibitors [203]	Mortality within a time frame of 28 days
*NCT* *05,341,609*	3	China	JT001 (VV116; remdesivir derivate)	nirmatrelvir/ritonavir	Inhibitor of RdRPs [204]	Time to sustained clinical recovery up to 28 days
*NCT* *04,315,948*	3	France	-Remdesivir -lopinavir/ritonavir-interferon beta-1a-hydroxychloroquine-standard of care -AZD7442	placebo	Inhibition of viral proteases (lopinavir/ritonavir) [201]	Percentage of subjects reporting each severity rating on a 7-point ordinal scale within a time frame of 15 days (a. Not hospitalized, no limitations on activities; b. Not hospitalized, limitation on activities; c. Hospitalized, not requiring supplemental oxygen; d. Hospitalized, requiring supplemental oxygen; e. Hospitalized, on non-invasive ventilation or high flow oxygen devices; f. Hospitalized, on invasive mechanical ventilation or ECMO; Death)
*NCT* *05,305,547*	3	USA	S-217622	placebo	M^pro^ inhibitor	Hospitalization from any cause or death from any cause

*RdRPs* RNA-dependent RNA polymerases, *IMPDH* inosine monophates dehydrogenase, *RT-PCR* reverse transcriptase-polymerase chain reaction, *CRP* C-reactive protein, *NMP* nucleoside monophosphates, *ECMO* extracorporeal membrane oxygenation

Second, the broader spectrum of oral antivirals would decrease the odds of resistance evolution in SARS-CoV-2. Some of these pharmaceuticals, differing in the mechanism of action, could be tested in combination, further reducing the risk of resistance and potentially increasing the efficacy of such therapies while decreasing their duration. At present, there is no clinical data on the combined use of nirmatrelvir/ritonavir and molnupiravir. However, it is doubtful if such a combination would be rational, given the fact that the former aims to prevent viral replication while the basis of the latter relies on direct interference with this process.

Third, both nirmatrelvir/ritonavir and molnupiravir require high dosing over the course of 5 days (Table 1), increasing the possibility that some doses will be missed. This may decrease the efficacy of treatment but also support the resistance evolution. It must be stressed that although the dosing of nirmatrelvir/ritonavir and molnupiravir may be challenging for some patients in the in-house setting, it is pivotal to complete the course of treatment according to the guideline. However, judging from the experience with antibiotic use, some patients who self-administer the oral COVID-19 antivirals may tend to cease their use as soon as an improvement is seen before completing the treatment protocol. The antivirals requiring lower doses and/or shorter duration of use may decrease the risk of such events.

Last but not least, the development of novel oral antivirals increases the odds that some could be repurposed if the next pandemic caused by a spread of RNA virus (be it coronavirus or not) occurs in the future. The threat of such a pandemic shall no longer be ignored given the present globalization, increase in livestock, the expansion of human settlements in more remote areas, and human contact with wild animals [195]. Research and development of oral antivirals for COVID-19 treatment may also lead to discoveries that may translate to the treatment of other viral diseases.

Table 4 summarizes over ten clinical trials initiated by the end of May 2022 and involving candidates for oral COVID-19 antivirals, which differ in mechanism of action. Six of them involve favipiravir, a selective inhibitor of viral RNA-dependent RNA polymerase and has already been approved for use against influenza in Japan [196]. The phase 3 trials on favipiravir involve patients with mild or moderate COVID19 and have similar primary outcomes, notably clinical improvement, with varying time frames across trials. In the Nepali (NCT04694612) and Hungarian (NCT04600999) trials, subjects are treated only with favipiravir, minimizing the confounding pharmaceutical factors. Among phase 2 trials, the favipiravir trial from Bangladesh (NCT04402203) and the AT527 trial (NCT04396106) from the USA are also the only ones where the oral antivirals are tested against the standard of care and placebo, respectively. There are also promising two oral SARS-CoV-2 M^pro^ inhibitor candidates in the advanced clinical studies: S-217622 (a noncovalent, nonpeptidic inhibitor of Mpro) which has been introduced in phase 3 clinical trial [197], and masitinib, previously authorized for treatment of mast cell tumors in dogs and evaluated in clinical trials for use in patients with asthma, certain cancers and neurological disorders, currently in phase 2 clinical trial for potential use in COVID-19 treatment [198] (Table 4).

## Ethical issues in clinical trials of oral antivirals in COVID-19

Several ethical issues can be identified concerning clinical trials of oral antiviral treatments for COVID-19. One such issue is the use of a placebo as a treatment comparator. Spławiński and Kuźniar (2004) assert that testing a new drug against a placebo in a superiority trial is ethically unacceptable despite being scientifically sound [205]. Meanwhile, it is argued that a placebo is ethically permissible when:

1. No proven effective treatment for the disease exists at the time of the study;
2. withholding treatment is posing negligible risks to study participants;
3. there are compelling methodological reasons for using a placebo, and withholding treatment does not pose a risk of serious harm to study participants;
4. there are compelling methodological reasons for using a placebo, while the research intends to develop interventions that can be implemented in the population from which trial participants are drawn, and the trial does not require participants to forgo treatment they would otherwise receive [206]

While some of these cases are easier to satisfy (e.g., case 1), others can prove harder to meet, particularly when it involves ascertaining minimal risk or harm. Since there is still limited knowledge about COVID-19 and the effects of experimental treatments, the risks of using a placebo as a treatment comparator are still high. Thus, similar to COVID-19 vaccine trials wherein "core ethical values strongly recommend that participants who had received placebo in COVID-19 vaccine trials should be promptly vaccinated after an interim analysis had proven that the vaccines are efficacious" [207], one could postulate that participants who were under the placebo arm (who often are representatives of a high-risk group of severe disease) should be given the COVID-19 oral antiviral treatment as soon as it was proven to be effective. However, this is virtually impossible since oral antivirals need to be given early after SARS-CoV-2 infection. Fortunately, there are two oral antivirals, i.e., nirmatrelvir/ritonavir and molnupiravir, clinically evidenced to be efficient in the treatment of SARS-CoV-2 and authorized in various world regions. Therefore, future clinical trials of oral antiviral candidates in COVID-19 treatment should include arms involving nirmatrelvir/ritonavir, molnupiravir or both instead of a placebo. This is already practiced, e.g., in the phase 3 trial of oral remdesivir derivate JT001 (VV116), safety and efficacy of which will be compared to nirmatrelvir/ritonavir (Table 4).

## Challenges of oral antiviral treatment of COVID-19

The authorization of the first-generation oral antivirals for COVID-19 treatment, i.e., nirmatrelvir/ritonavir and molnupiravir, is undoubtedly a step forward in better managing SARS-CoV-2 infections. However, their use can be associated with a number of challenges:

1. The use of nirmatrelvir/ritonavir is known to interact with various medicines which are routinely used in individuals representing high-risk groups in COVID-19. Molnupiravir appears to be superior in this regard as no known interactions have been identified so far. However, one needs to consider that various uncertainties exist, and real-world experience may unfold additional limitations of oral antivirals due to their adverse interactions with other medicines [208].
2. Oral antivirals are also intended for use outside the clinical setting, which can decrease the pressure on healthcare units but is also associated with less control over the treatment course and patients’ adherence to dosing protocol. This may be particularly challenging because both first-generation oral antivirals in COVID-19 require a high dosing regimen for 5 days. At the same time, missing a dose is a relatively common phenomenon, even in therapies such as highly active antiretroviral therapy [209, 210]. Adherence to recommendations is pivotal in decreasing the risk of resistance evolution, and in the case of molnupiravir, the rise of non-lethal mutations with the unknown outcome for SARS-CoV-2 fitness.
3. It is pivotal to initiate the administration of oral antivirals no later than five days after the onset of COVID-19 symptoms. There is a risk that in a real-world setting, many patients will start these therapies too late if one considers the lag that may occur between the development of first symptoms, making a decision to test toward SARS-CoV-2 infection, receiving a result confirming the infection, consulting a medical doctor, receiving a prescription, and obtaining the pharmaceutical.
4. Resistance to oral antivirals may arise over time, more likely in the case of nirmatrelvir/ritonavir, which is a combination of two protease inhibitors. The evolution of resistance to viral protease inhibitors was already evidenced for other viral infections such as HIV-1 and HCV [211].
5. Since ritonavir, a component of paxlovid formula, is widely used to control HIV infections, manufacturing paxlovid might affect the availability of ritonavir for HIV patients [212].
6. The outcomes of oral antivirals in different patients may be significantly heterogeneous with confounders including age, BMI status, and comorbidities. Similar to various other pharmaceuticals, there might be a need to optimize the dose in various groups of patients [213]. The potential need to increase the dosing in some individuals may limit the use of antivirals due to adverse effects or drug interactions, while a decrease in dosing may potentially lead to an efficacy reduction.
7. The use of oral antivirals for COVID-19 treatment in the pediatric group may be limited. Molnupiravir is contradicted in children due to potential bone and cartilage toxicity. The use of nirmatrelvir/ritonavir is not authorized in pediatric patients younger than 12 years of age or weighing less than 40 kg. The clinical trial involving non-hospitalized children with COVID-19 aged 6–12 who are at risk of progression to severe disease is ongoing (NCT05261139; Table 2). However, due to numerous interactions of nirmatrelvir/ritonavir with other pharmaceuticals, there might be a need to develop novel oral antivirals for pediatric patients.
8. The first-generation oral antivirals for COVID-19 treatment are expensive therapies [214], likely limiting their availability even in developed countries. There is a risk their accessibility in low-income countries will be poor, similarly to COVID-19 vaccines [25, 40, 219].
9. Similarly to the COVID-19 vaccines, the oral antivirals for COVID-19 treatment may be subject to hesitancy. This is because the COVID-19 pandemic has seen an unprecedented flood of misinformation spread worldwide and a rise of various conspiracy theories that undermined the trust in health authorities [26, 215, 216]. In the case of some individuals, a belief in conspiracy theories can be emotionally rooted to the point that even personal experiences with COVID-19 are insufficient to change one’s perception [217].

It should be stressed that the challenges mentioned above may be partially overcome by the continuously increasing experience with nirmatrelvir/ritonavir and molnupiravir use, authorization of novel oral drugs for COVID-19 treatment, combined use of more than one anti-SARS-CoV-2 treatment, and development of generic, less expensive versions of oral antivirals. In turn, the hesitancy to use the oral antivirals require accurate and honest communication with the patient and an explanation of the risks and benefits associated with drug administration.

## Conclusions

The odds for eradication of SARS-CoV-2 in the near future are very low. It is reasonable to assume that COVID-19 is here to stay, making it necessary to adapt to its existence to prevent healthcare systems from being overwhelmed. This adaptation should involve various elements, including (i) tracking of SARS-CoV-2 evolution through genome sequencing, (ii) pursuing vaccinations, (iii) developing and testing of vaccines optimized toward various viral variants and inducing prolonged immunity, (iv) research on pan-coronavirus vaccine, (v) swift implementation of non-pharmaceutical prevention measures during periods when an increased number of infections can be expected (e.g., between autumn and spring in the temperate zone), (vi) optimizing treatment protocols for various groups of hospitalized patients, and (vii) making oral COVID-19 antivirals available to decrease the risk of hospitalization in infected, high-risk individuals. Currently (mid-2022), two oral antivirals are available, nirmatrelvir/ritonavir and molnupiravir, that differ in their mechanism of action. A broader spectrum of oral antiviral pharmaceuticals should be available in the future. The priority is to make these treatments accessible globally, including in the low-income countries, and use them according to recommendations to decrease the odds of resistance evolution.

## Data Availability

Not applicable.
