# Correction: Oral antiviral treatments for COVID-19: opportunities and challenges

**DOI:** 10.1007/s43440-022-00410-y

**Published:** 2022-09-23

**Authors:** Laila Rahmah, Sunny O. Abarikwu, Amanuel Godana Arero, Mickael Essouma, Aliyu Tijani Jibril, Andrzej Fal, Robert Flisiak, Rangarirai Makuku, Leander Marquez, Kawthar Mohamed, Lamin Ndow, Dorota Zarębska-Michaluk, Nima Rezaei, Piotr Rzymski

**Affiliations:** 1 School of Medicine, Tehran University of Medical Sciences, Tehran, Iran; 2 Universal Scientific Education and Research Network (USERN), Jakarta, Indonesia; 3 Department of Biochemistry, University of Port Harcourt, Choba, Nigeria; 4 Universal Scientific Education and Research Network (USERN), Choba, Nigeria; 5 Cardiac Primary Prevention Research Center, Cardiovascular Diseases Research Institute, Tehran University of Medical Sciences, Tehran, Iran; 6 Universal Scientific Education and Research Network (USERN), Addis Ababa, Ethiopia; 7 Department of Community Nutrition, School of Nutritional Sciences and Dietetics, Tehran University of Medical Sciences, Tehran, Iran; 8 Nutritional and Health Team (NHT), Universal Scientific Education and Research Network (USERN), Tehran, Iran; 9 Universal Scientific Education and Research Network (USERN), Accra, Ghana; 10 Department of Population Health, Division of Public Health, Wroclaw Medical University, Wroclaw, Poland; 11 Collegium Medicum, Warsaw Faculty of Medicine, Cardinal Stefan Wyszyński University, Warsaw, Poland; 12 Integrated Science Association (ISA), Universal Scientific Education and Research Network (USERN), Poznań, Poland; 13 Department of Infectious Diseases and Hepatology, Medical University of Białystok, Białystok, Poland; 14 Universal Scientific Education and Research Network (USERN), Harare, Zimbabwe; 15 College of Social Sciences and Philosophy, University of the Philippines Diliman, Quezon City, Philippines; 16 Education and Research Network (USERN), Universal Scientific, Quezon City, Philippines; 17 Universal Scientific Education and Research Network (USERN), Manama, Bahrain; 18 National Health Laboratory Service, Kotu, Gambia; 19 Universal Scientific Education and Research Network (USERN), Banjul, Gambia; 20 Department of Infectious Diseases, Jan Kochanowski University, Kielce, Poland; 21 Research Center for Immunodeficiencies, Children’s Medical Center, Tehran University of Medical Sciences, Tehran, Iran; 22 Department of Immunology, School of Medicine, Tehran University of Medical Sciences, Tehran, Iran; 23 Network of Immunity in Infection, Malignancy and Autoimmunity (NIIMA), Universal Scientific Education and Research Network (USERN), Tehran, Iran; 24 Department of Environmental Medicine, Poznan University of Medical Sciences, Poznań, Poland; 25 Department of Internal Medicine and Specialties, Faculty of Medicine and Biomedical Sciences, University of Yaoundé I, Yaoundé, Cameroon; 26 Universal Scientific Education and Research Network, Yaoundé, Cameroon

## Correction: Pharmacological Reports 10.1007/s43440-022-00388-7

The original version of this article unfortunately did not list Mickael Essouma as a co-author who has been involved in the drafting and approving of the manuscript.

 A correct list of authors is as follows: Laila Rahmah, Sunny O. Abarikwu, Amanuel Godana Arero, Mickael Essouma, Aliyu Tijani Jibril, Andrzej Fal, Robert Flisiak, Rangarirai Makuku, Leander Marquez, Kawthar Mohamed, Lamin Ndow, Dorota Zarębska-Michaluk, Nima Rezaei & Piotr Rzymski.

Along with that “Author contributions” was also updated. The original article has been corrected.

